# Response of Ruderal Species Diversity to an Urban Environment: Implications for Conservation and Management

**DOI:** 10.3390/ijerph15122832

**Published:** 2018-12-12

**Authors:** Peipei Guo, Fang Yu, Yuan Ren, Dong Liu, Jia Li, Zhiyun Ouyang, Xiaoke Wang

**Affiliations:** 1 Center for Environmental Risk and Damage Assessment, Chinese Academy for Environmental Planning, Beijing 100012, China; guopp@caep.org.cn (P.G.), yufang@caep.org.cn (F.Y.); 2 Research Center for Eco-Environmental Sciences, CAS, Beijing 100085, China; zyouyang@rcees.ac.cn; 3 State Key Laboratory of Subtropical Silviculture, Zhejiang A&F University, Hangzhou 311300, China; renyuan@zafu.edu.cn; 4 Nanjing Institute of Environmental Sciences, Ministry of Environmental Protection, Nanjing 210042, China; liudong@nies.org; 5 Lanxi Environmental protection monitoring station, Lanxi 321100, China; lijiazhelin2@126.com

**Keywords:** origin, invasive, pollen allergenic, chorological types

## Abstract

Anthropogenic activities have weakened the invasion of ruderals and increased the number of non-native species in urban areas. Ruderals are an important component of urban plant diversity and are of great significance to the sustainable development of urban green space. We used the tessellation–randomized plot method to examine the composition and biodiversity of ruderal species among urban land use types (LUTs) in the built-up areas of Beijing. Soil samples from the surface to a depth of 10 cm were taken from each site to examine the impact of soil characteristics on ruderal species diversity. Results showed that a total of 120 ruderal species were observed, including 71 native and 49 non-native species. Among them, 90% were identified as Cosmopolitan. Native species accounted for the majority of ruderals across all the eight LUTs. Institutional, residential, and woodlot areas with coarser management had higher ruderal species richness than did commercial areas and roadside lawns. Allergenic species showed the highest proportions in municipal parks, and invasive species accounted for 20% of all ruderal species. Ruderal species diversity was related to distance from the urban center, pruning intensity, and soil characteristics. These results suggest that with ruderals playing an important role in urban grass species diversity, there is tremendous potential for more native species in Beijing lawns, which would contribute substantially to increasing the ecological system’s functional benefits. Ruderal species accustomed to the causal processes and environmental conditions of different LUTs should be used and conserved properly to improve the harsh conditions of different LUTs and to sustain ecosystem health.

## 1. Introduction

Ruderals are an important component of the urban green space, are distributed widely in and around cities, occur at no financial cost, and are always appropriate in urban conditions. However, the use of ruderals for landscaping purposes in urban green spaces has been largely ignored.

Ruderals are a kind of spontaneous vegetation and have great significance for biodiversity in urban areas. Ohsawa (1988) defined ruderals as a vegetation type that invades cities and becomes widely distributed without relying on human intervention [1]. They have a survival strategy which can adapt to local habitats and resist intense human interruption. Spontaneous vegetation plays an important ecological and aesthetic role in urban sustainable landscape development according to a survey of ruderals in Romania [2]. Some research has suggested that ruderals could contribute to valuable ecosystem services [3], such as reducing temperature, providing a habitat for wildlife, reducing runoff, and carbon sequestration. The management of spontaneous vegetation in urban areas to increase its ecological and social value is a sustainable strategy for the development of urban green spaces [4]. Urbanization has been an important driver of biodiversity loss [5], especially for ruderal species with low economic value, which often indicates dereliction. The relatively few non-native species thriving in well-managed urban grass landscapes tend to predominate [6]. Some of the most common species of vegetation in Xi’an were considered invasive [7]. The invasion of exotic plant species in Hong Kong was mainly confined to habitats that were subjected to long-term human disturbance [8]. The grassland species richness dropped by 30–50% during a fifty-year period in Northern Germany [9]. At the same time, because of the high heterogeneity of urban space, a large number of local and exotic species can exist in urban areas, leading them to be rich in plant species [10,11]. 

Although lawns are one of the most popular green space types for urban residents, it was proved that well maintained lawns provide bad conditions for some species [12]. Currently, most urban lawns are of a single species with a high economic cost. There is great scope for these lawns to become more biodiverse by conserving and introducing native and ruderal species. Recently, some European researchers had suggested that old patches of spontaneous vegetation in cities should be conserved because they play an important role in producing and maintaining urban biodiversity [13,14]. A variety of natural species habitats (vacant space, abandoned industrial areas, parking lots, and along railway lines, highways, and other roadways) often support highly diverse plant species [3]. Compared with traditional lawns, ruderal-like vegetation increases species diversity and reduces maintenance costs [15]. Some research has dealt with design using spontaneously occurring species in urban greening [16]. Research of a park in Paris showed that local residents preferred to consider the management of wild plants from the perspective of cohabitation rather than abandonment, which may indicate that people desire more natural landscapes [17]. 

While urban spontaneous vegetation is usually considered as adding no value, more and more studies are providing an in-depth understanding of various ecosystem services which are not captured in the current urban ecosystem models [3]. The study of urban ecology in North America usually focuses on the residual natural spaces in cities rather than the unique urban plant communities [18]. Characteristics of urban vegetation including forests [19] and spontaneous vegetation [20] were investigated in the Halifax area of Canada. Recently, research on ruderals has been mostly focused on plant diversity and composition. Jim and Chen conducted surveys of the spontaneous arboreal flora on buildings of different configurations and ages in the densely populated city of Hong Kong [21]. A study in Harbin suggested that grass species with a short life span, a high drought tolerance, fast growth rates, and high seed yields were more preferred in urban habitats [22]. A study in upland grasslands of the southern part of Czech Republic analyzed the relationship between environmental factors and species richness and composition [23]. The impact of human activities on the biodiversity of spontaneous plants upstream of the Lumbardh, Prizren, Kosovo, was analyzed, and the suggestion was made to conserve and protect spontaneous plants [24]. Management and assessment of medicinal plants from spontaneous flora was conducted in the republic of Moldov [25].

More and more people have moved into cities with highly modified, human-dominated environments where nature is considered as expendable and the ecological processes that sustain life have almost disappeared. Expenditures on green spaces are often considered to be a luxury and are often the first items to be eliminated from municipal budgets in times of economic tension. More and more people cannot correctly identify common ruderal grass species with local distributions, and the relationships between urbanization, habitat loss, and species reduction are usually neglected [26]. Urban plant composition and vegetation types are mostly decided by urban managers. Although biodiversity is high in certain parts of urban areas, species extinction has intensified worldwide. The native species with low aesthetic value are often ignored and neglected (removed) almost to the fringe of extinction; these native grass species play important roles for local ecosystem health. Conservation of ruderals relies on reasonable and effective use, with the aim to make them accepted and identifiable by people. Aesthetics is just one aspect; how to manage them to increase their ecological and socio-economic value should be further explored. More efforts should be made to make the natural landscape the foundation of people’s lives.

In this study, we quantify ruderal diversity across eight land use types (LUTs) in order to provide scientific advice and a theoretical base to the designed integration of existing ruderals in urban areas and to contribute to creating a sustainable, ecologically functioning urban landscape. Although this study was carried out in Beijing, we hope that our research findings will be of more general relevance to urban green spaces throughout the northern temperate area. We sought answers to the following questions for a sample of plots in eight LUTs in built-up areas of Beijing: (1) How do ruderal compare, in terms of composition, origin, chorological types, invasion, pollen allergenicity, and functional traits among the eight LUTs? (2) Are the compositions of grasses determined by social economic factors (human selection, management policy), influenced by regional factors (environmental filter, e.g., climate, distances from urban center, and soil quality), or determined by ruderal characteristics (reproductive pattern, life form)? (3) What are the implications for the conservation and management of ruderals in urban areas?

## 2. Study Site and Methods

### 2.1. Study Site

The study was carried out within the fifth ring road in the built-up area of Beijing (39°45′–40°2′ N, 116°11′–116°33′ E), which is the capital of China, located in the north of the country. The terrain of the study area was flat with an area of 670 km^2^. The average altitude is about 50 m. The city lies in a warm, temperate, semi-humid climate zone. The average annual temperature is about 11–12 °C, and the annual precipitation is 500 mm. 

### 2.2. Sampling Design and Field Investigation

Our research of ruderals in Beijing’s built-up areas was based on the tessellation–randomized plot method [19,27]. The eight primary LUTs were defined as institutional, residential, commercial, community park, municipal park, woodlot, roadside, and riverside. One to three LUTs were selected randomly in each 2 × 2 km grid square of the Beijing city map, and two or three sample plots of 20 × 20 m were chosen randomly in each LUT. Then, three to five subplots of 1 × 1 m quadrats were randomly chosen in each sample plot. About 1046 subplots were sampled at 300 random points throughout the study area. SPOT remote sensing images with a resolution of 10 m were used to stratify the field samples and to determine the center point of each sample plot. Garmin 60CXs GPS (Garmin International, Inc., Olathe, KS, USA) was used to locate the plot centers. 

For ruderal plots, the average height of each species was measured and recorded. The coverage of each species was estimated by sight. The number of individuals of each species was counted and recorded. In cases where individuals of species in a plot were too abundant, individuals in a subplot with a smaller size (0.1 × 0.1 m) were counted, and this count was used to calculate the total number of individuals in the entire plot.

Soil core samples (2.54 cm in diameter) were impacted from the surface to a depth of 10 cm by using an impactor corer; five locations were randomly selected at each plot. The soil samples were sieved (0.15 mm mesh). A total of 300 soil samples from 300 sites were taken and measured for nitrogen (N), carbon (C), and the nutrients phosphorus (P), potassium (K), magnesium (Mg), and calcium (Ca). Total C and N were analyzed using Elementar (vario EL III, Frankfurt, Germany). The nutrients were extracted using a microwave extraction system (MARS, CEM Corporation, Matthews, NC, USA), and the concentrations were then analyzed using an Inductively Coupled Plasma Optical Emission Spectrometer (ICP-OES, PRODIGY, LEEMANS, Hudson, NH, USA).

### 2.3. Species Origin

Plant species were divided into two categories: native and non-native. The non-native species were further classified according to their place of origin—originating from within or outside the Beijing area. We then placed each plant species into one of three origin classes: (1) species that were identified as species originating in Beijing (native); (2) species that were native to China and identified as non-native to Beijing (extralimital native); and (3) species that were non-native to China (exotic). Plant species were identified according to the related preliminary information and to their distribution throughout the country [28,29,30]. 

### 2.4. Chorological Types

The chorological spectrum refers to the geographical distribution of specific genera in the world [31]. The species surveyed were analyzed according to the classification standard of plant chorological types by Wu (1991), with the following elements: Cosmopolitan, Pantropic, Old World Tropics, Tropical Asia and Tropical Australasia, Tropical Asia (Indo–Malesia), North Temperate, and so on.

### 2.5. Invasive Species

Invasive species were identified based on “Biological Invasions: Color Illustrations of Invasive Alien Plants in China” [32], “Exotic Plants in China” [28], and the references therein [33]. 

### 2.6. Pollen-Allergenic Species

Pollen allergenic plants species in Beijing’s built-up area were determined mainly based on the reference “Airborne pollen allergenic investigation in China” [34].

### 2.7. Socioeconomic Variables 

Data on years since the LUT was built, house prices, distances from the urban center, and the greening rate for each sample site were collected. House prices and greening rate data were derived from websites reporting housing information. Other greening rate data were acquired from the “Beijing Statistical Yearbook 2014” [35].

### 2.8. Data Analysis

Ruderal diversity was compared among eight LUTs. Variables representing the main geographical, environmental, and socio-economic characteristics of the study site were chosen (distance from urban center, soil C and N concentration, C/N ratio, soil nutrients, number of years since LUT built, population density, and median house price). The species richness of different LUTs was compared using one-way ANOVA and LSD (least significance difference test); when the *p*-value was less than 0.05, the difference was considered to be significant. SPSS 20 (IBM Corp., Armonk, NY, USA) was used to conduct the statistical tests. Canoco 4.5 was used to conduct the canonical correlation analysis (CCA) of species richness and socioeconomic variables [36].

## 3. Results

### 3.1. Ruderal Species Diversity

**Figure 1:**
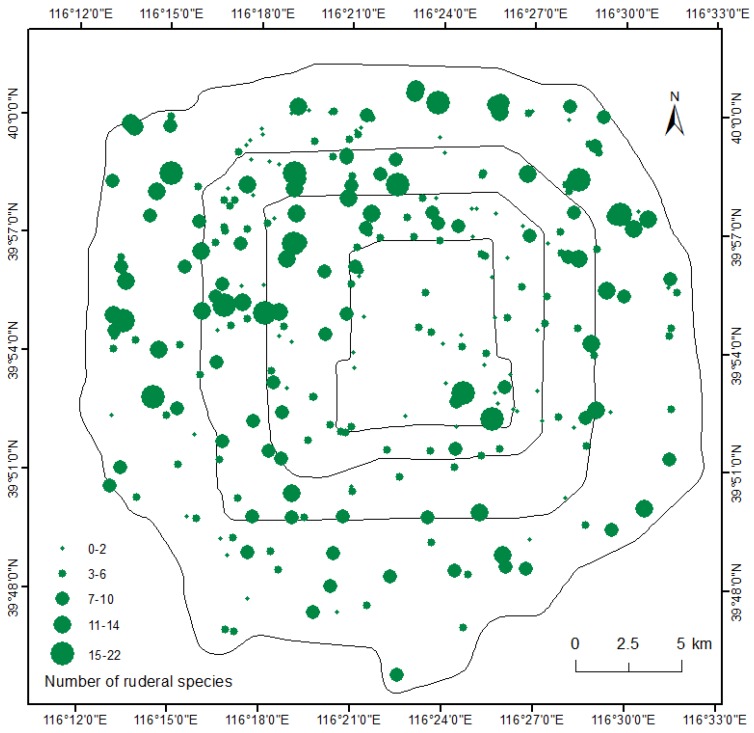
Number of ruderal species in the built-up areas of Beijing.

A total of 120 ruderal species belonging to 30 families and 75 genus were recorded in 1046 sample plots in the built-up areas of Beijing. There is an obvious variation in the spatial distribution of ruderal species richness across the urban landscape (Figure 1). The north area showed higher richness than did the south area.

**Figure 2:**
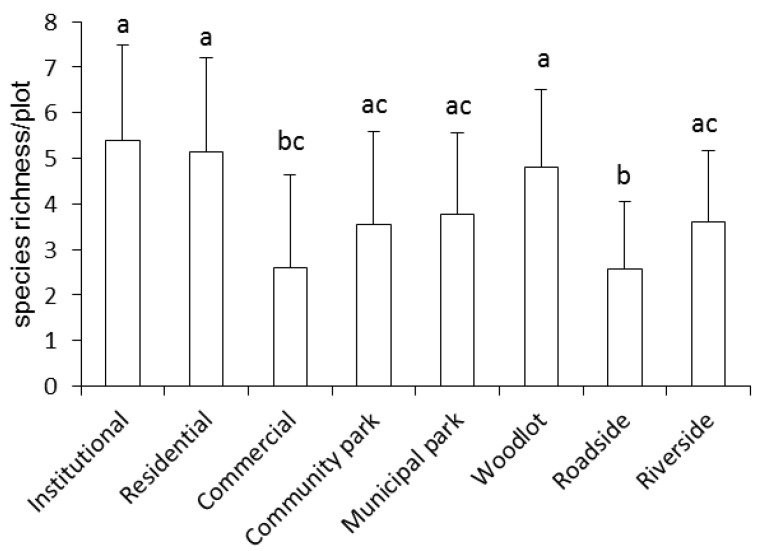
Ruderal species richness in different land use types (LUTs) in the built-up areas of Beijing.

Figure 2 presents the mean ruderal species richness for each LUT. Institutional, residential, and woodlot types had higher ruderal species richness than did commercial and roadside (*p* < 0.05). 

The ruderals with the highest frequency in the built-up areas of Beijing were *Oxalis corniculata* and *Setaria viridis*, with frequencies of 36% and 26%, respectively. The results of different LUTs showed that *Oxalis corniculata* had the highest frequency of 36.3% in institutional areas, while residential, commercial, municipal park, roadside, and riverside areas consisted mostly of *Setaria viridis*. The frequency of *Taraxacum mongolicum* was the highest in community parks, and woodlots consisted mainly of *Chenopodium glaucum*, with a frequency of 67.7% (Table 1).

The 20 most common species appeared in about 44% of all subplot records. A large proportion of species recorded were infrequent (51% occurred at two sites or fewer).

### 3.2. Species Origin

**Figure 3:**
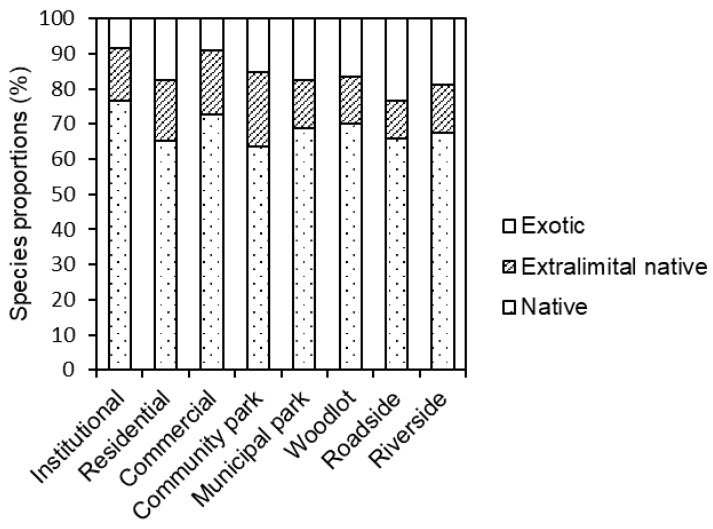
Ruderal species origins in the eight LUTs in the built-up areas of Beijing.

In this study, 71 native species, 26 extralimital native species, and 23 exotic species were observed in the built-up areas of Beijing. The native ruderal species made up the highest proportion of species for all eight LUTs—all higher than 60%. The percentage of native species at each LUT ranged from around 64% (community park) to 77% (institutional), extralimital native species ranged from 11% (roadside) to 21% (community park), and exotic species ranged from 8% (institutional) to 23% (roadside) (Figure 3).

**Figure 4:**
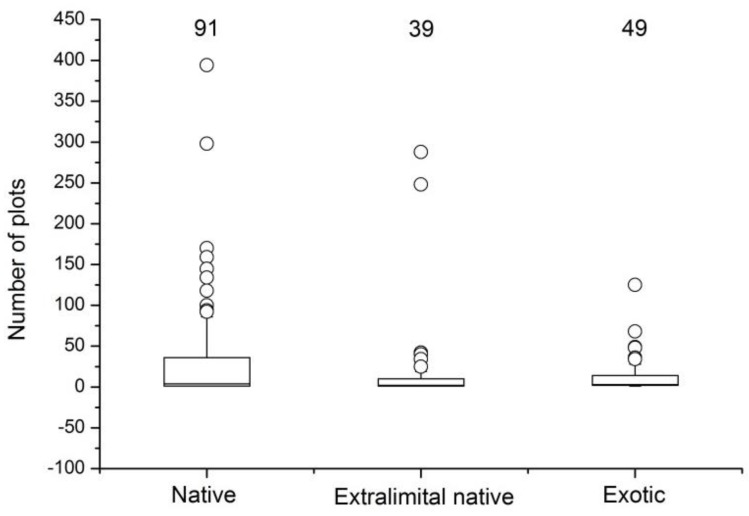
The frequency of occurrence per plot of native, extralimital native, and exotic species. Number of species is provided at the top of each box. Outliers are represented by hollow dots, and whiskers represent data within 1.5 times the interquartile range of the upper and lower quartiles.

Native species were distributed more widely than the extralimital native and exotic species (Figure 4). Native plant species made up the highest proportion in the survey of Beijing ruderals recorded in 1046 plots: native species constituted 51% of the 120 species, followed by 27% exotic species and 22% extralimital native species.

About 73% of the 30 most widespread species, measured by frequency of occurrence in survey plots, were native species; the remaining 27% were non-natives. The most widespread native species, *Setaria viridis* (present in 394 sites), was much more widespread than the most widespread extralimital native species, *Chenopodium glaucum* (248 sites), and exotic grass species, *Eleusine indica* (125 sites). 

**Figure 5:**
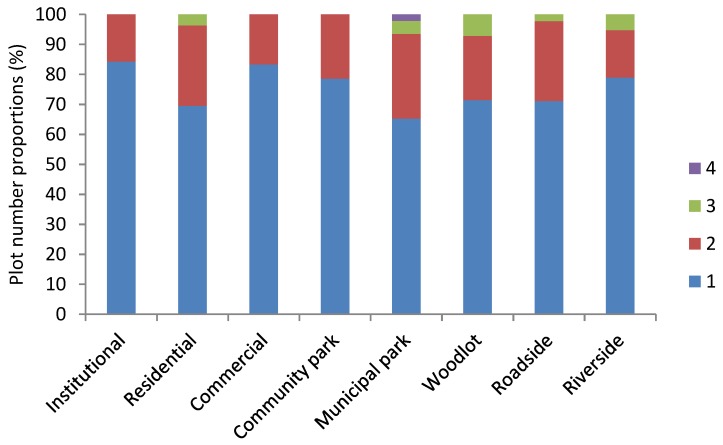
Distribution of invasive ruderal species by number in each plot for different LUTs.

There were between one and four invasive species in each plot; plots with one invasive species were most common, accounting for 72% of all plots that contained invasive plants (Figure 5). For institutional areas, about 84% of plots had one invasive species—the highest among the eight LUTs. The invasive species were widely distributed in the urban area and were reasonably applied under the control of human beings.

### 3.3. Pollen Allergenic Species

There were 12 pollen allergenic species documented in the survey of the built-up areas of Beijing. Pollen allergenic species appeared in 54% of the sample plots. The species recorded with the highest frequency was *Setaria viridis*, followed by *Chenopodium glaucum*. Plots containing pollen allergenic species occurred most commonly in woodlots, where they made up 86% of plots; the main species were *Setaria viridis* and *Chenopodium glaucum*, accounting for 67% of all pollen allergenic species. Commercial land showed the lowest proportion of plots with pollen allergenic species, at 31%.

**Figure 6:**
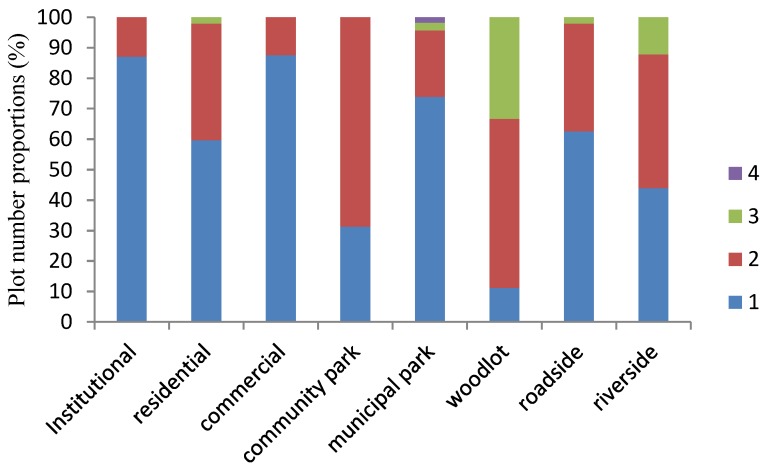
Distribution of pollen allergenic ruderal species by number in each plot for different LUTs.

Plots containing one pollen allergenic species occurred most commonly in the various LUTs, except in community parks and woodlots (Figure 6). The surveyed plots containing two pollen allergenic species accounted for 69% of plots in community parks, followed by 56% in woodlots. The pollen allergenic species made up high proportions of the species in some LUTs, such as community parks and woodlots. The number and distribution of pollen allergenic species should be controlled for the health of urban residents.

### 3.4. Impact Factors

**Figure 7:**
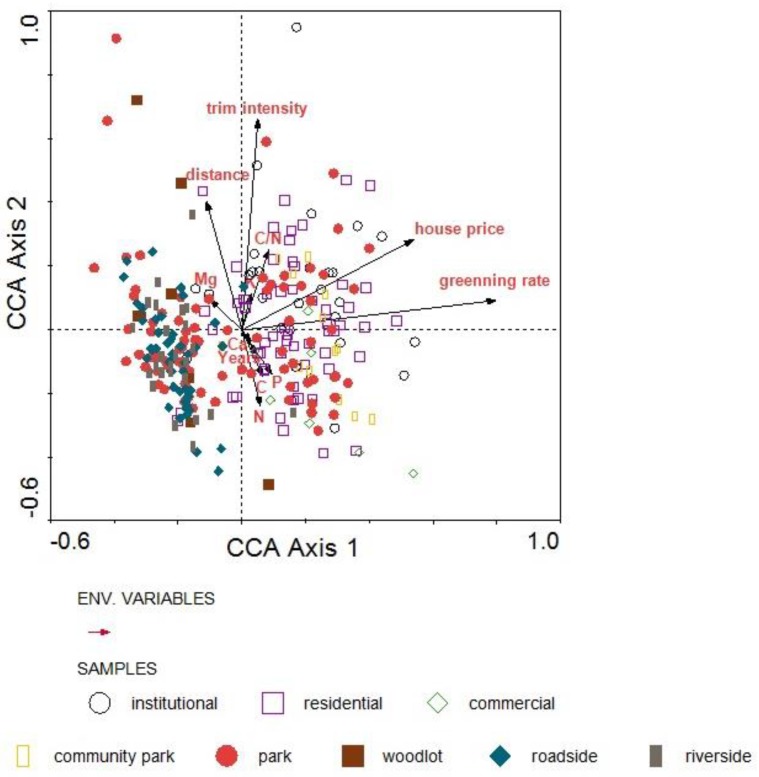
Canonical correspondence analysis (CCA) of plots and the environmental variables.

Canonical correlation analysis (CCA) resulted in a bi-plot of ordination space showing ruderal species richness in relation to soil properties and socioeconomic variables (Figure 7). Results showed that Axis 1 was related to the greening rate; the canonical correlation coefficient was 0.78. Axis 2 was related to the distance from the urban center, the pruning intensity, and soil properties. The scatter plot showed that there was a significant differentiation of species richness among LUTs. Institutional plots were relatively concentrated in the first quadrant, showing a positive relationship with pruning intensity, soil K content, and C/N ratio. Residential plots were concentrated in the first and the fourth quadrant, showing a positive relationship with greening rate and house prices. Roadside and riverside plots were both focused in the third quadrant with no significant relationship with soil characteristics, but both had a significantly negative correlation with house price and greening rate. Ruderal abundance is high in areas with low house prices and low greening rates. A lack of management in these areas may lead to higher ruderal abundance.

Distance from the urban center was positively correlated with pruning rate, the content of Mg and K, and the C/N ratio, while it was negatively correlated with the number of years since the LUT was built and the content of C, P, and Ca. The greater the distance from the urban center, the higher the pruning intensity. This is because parks far away from the urban center often lack human management to sustain the lawn landscape, and herbicides are adopted to eradicate all ruderals, leading to high intensity ruderal removal. There was a significantly positively relationship between house price and greening rate. The CCA results explain the reason for higher ruderal richness in the north area, as compared with the south area: The number of municipal parks is higher in the north area, and their management and maintenance is more delicate, which leads to greater ruderal species richness. Further, from the aspect of the theory of the “luxury effect” [18], the higher house price in the north areas also contribute to higher species richness.

## 4. Discussion

### 4.1. Composition and Diversity of Ruderal Species

Ruderal species richness was high across all eight LUTs, but familial richness was not. Relatively few plant families represented the majority of species occurrences. We observed 120 ruderal species belonging to 30 families and 75 genus during plot sampling, referring to 13 chorological types and 6 subtypes. Chen (2005) observed 107 grass species of 89 genus and 32 families belonging to 14 chorological types and 5 subtypes in downtown Shanghai [37]. Li (2007) reported 124 species of plants in built-up areas of three northeast provinces of China [38]. The most frequent ruderal families in the current study were Gramineae and Asteraceae. Poaceae and Asteraceae accounted for 16% of all surveyed species. These common species were widely adopted as afforestation trees in the built-up areas of Beijing, mainly due to their pioneer characteristics, fast establishment, and landscaping aesthetics. Poaceae possesses highly evolved inflorescence and an effective anemophilous pollination mechanism. Asteraceae has the highest number of species among vascular plants and is also one of the most evolutionarily advanced families. It contains some characteristics that are easily diffused, for example, a high reproduction potential, diverse reproductive patterns, and seeds that are fit for wind dispersal. Fabaceae has a unique nitrogen fixing capacity and possesses superiority in resources through nutrient accumulation. The principal lawn families in northern temperate conditions are Poaceae, Asteraceae, Fabaceae, Cyperaceae, Oxalidaceae, Apiaceae, Scrophulariaceae, Plantaginaceae, Caryophyllaceae, Ranunculaceae, Juncaceae, and Brassicaceae [40]. The composition of Beijing lawns is very similar to lawns in northern temperate conditions worldwide, reflecting global homogenization of lawn flora.

Ruderals account for a large portion of urban flora because they are typically tolerant of disturbance [10] and are generally wind-dispersed (*Taraxacum mongolicum*) [6]. Urban spontaneous vegetation is dominated by closely related species that share functional traits that make them more suited to urban conditions [3]. This study supports this by showing that species from families such as Gramineae and Asteraceae were more numerous than species from other plant families. These families have many traits that make them well suited for urban conditions, such as minor seeds, the ability to germinate immediately, and rapid growth.

The exotic continental ruderal species mainly originated from America. This is because the two areas have a similar latitude, climate, and biota. Species migrating mutually between these two areas can quickly adapt to the local habitat and increase their colonization opportunity. Furthermore, frequent trade contacts also favor species introduction.

Ruderals in Beijing’s built-up areas do vary in composition among different LUTs. Roadside lawns had lower species richness than other LUT lawns, except for commercial lawns. Lawns in commercial and roadside areas are usually seeded and maintained with non-native grass, so a low percentage of ruderal species was expected. These differences may reflect the effects of “lawn care” such as mowing, irrigating, removal of clippings, and litter accumulation more than environmental and social variables [39]. Lawn composition varies according to age, proximity to seed sources, and the degree of management but will also depend on both natural and managed environmental conditions [39].

Native species accounted for the majority of ruderals. Our results indicated that the percentage of native species in lawn ruderals is the highest in Beijing’s built-up areas (higher than 60%). This is similar to the results of a study of ruderals in Beijing’s built-up area in 2007, which showed that 69% of ruderals were of native species [40]. Research on lawn flora in the tropics–subtropics or arid environments showed similar results (e.g., Bolivia, 80%), and a lower percentage was found in temperate Chile, Southern Australia, and New Zealand (20, 11, and 19%, respectively) [41]. Native species play a more important role in ecological balance than do alien species [19], and wild animals prefer a habitat with native species [42]. Therefore, more native species should be applied in the restoration of urban green spaces [43]. 

When introducing ruderals into urban lawns, more attention should be paid to the threat of invasive species. While the existence of exotic species in urban ruderal habitats increases the local biodiversity, it also carries a risk of invasion. Some research has showed that an invasive plant can exert significant negative effects on native plant communities, including a reduction in native plant biomass and a shift in native species composition due to the invasion [44]. Plant community diversity and native plant abundance were found to decline with increasing abundance of an exotic annual grass [45]. Exotic species pose some risk of becoming invasive, potentially causing severe damage to native species and local habitats.

A total of 31 invasive ruderal species were identified in Beijing’s built-up area; this is similar to the findings of a plot survey in 2003 and 2007, which documented 33 invasive species including 2 tree species and 31 grass species [40]. *Chenopodium glaucum* and *Eleusine indica* had become predominant species in the study area, and some other species had become distributed in all LUTs. These invasive species may reproduce prolifically and exert a great impact on the urban habitat as they lack enemies in this environment [46]. Further study should be carried out on the invasiveness of these species, and effective measures should be taken to control them. 

### 4.2. Recommendations on Management

Human selection and environmental filtering were two main factors that contributed to the differences in ruderal diversity among LUTs. The survival or elimination of species in urban areas is the response to disturbance and environmental constraints. Our result showed that the distribution of ruderal species was related with soil nutrients and human interruption. Nutrient accumulation and physical interruption were main characteristics of plant invasion under human influence. Ruderals in Beijing’s built-up areas were dominated by species with short life cycles (annual, biennial, and annual and biennial). These plants had a high growing rate and early maturation, and they therefore had more survival opportunities in a new place. Deterioration of soil conditions and human interference mainly accounted for the distribution patterns of urban ruderals.

The ruderal species composition was different among LUTs for the reason that management intensity and frequency were different among LUTs. The municipal parks had regular maintenance. However, the municipal parks were often open for free, had a large area, and had multifarious service objectives; these produced additional difficulties for maintenance and management. In the survey, some park lawns showed serious degradation. Residential areas showed obvious polarization. Some high-grade residential areas had well-maintained landscaping, while some old residential areas often showed degraded lawns with weeds cluttering the distribution. 

On the whole, the current urban lawn building mode is unsustainable. When the original lawns with single-species composition become degraded as a result of environmental disturbance, lawns are often restored by purchasing and planting new turf. In this way, the original land undergoes continual destruction. Under proper control and guidance, the invasion of ruderals can have a positive effect on the original lawn and play a constructive role in lawn restoration. In such a case, ruderals in urban areas could be the sustainer of lawn landscapes and could provide green coverage and a pleasing view. Therefore, ruderals in urban lawns should not be discarded completely but should be saved and trimmed with the lawn grass to maintain the integrity of the lawn landscape. In addition, the increase in the biodiversity in the grass layer could make the community structure complex enough to resist environmental interference and other invasions. 

To obtain a meaningful protection strategy, we must explore mechanisms for encouraging multiscale, complementary “ruderal-friendly” management of urban lawns. We are currently witnessing ruderal eradication in urban green spaces, such as artificial weeding or weed elimination by chemical herbicides. The cumulative outcome of different management styles is detrimental to the native ruderal species diversity in urban lawns.

Lawns are the dominant green space in urban areas, and more attention should be paid to their establishment and maintenance. The native ruderal species were particularly adapted to the environment, and they could be more environmentally friendly and cost-effective, with less irrigation, fertilizer, herbicides, and mowing required [47]. Introducing native ruderal species into urban lawns also provides an opportunity for nature conservation in urban areas. The most effective (and cheapest in the long term) strategy is to preserve and introduce as many native grass species as possible. 

Options for incentivizing turning urban green space into “ruderal-friendly” lawns fall into two categories: (1) top-down regulation and (2) bottom-up personal initiatives. Top-down approaches refer to public education on and the popularization of biodiversity and ecosystem services and government grants for sympathetic management. For ruderal and wildlife conservation purposes, tax incentives and subsidies are an increasingly popular tool. For a bottom-up strategy, the reservation or application of ruderal species in private gardens is important for introducing ruderal species into urban green spaces. 

## 5. Conclusions

Ruderals contribute to the biodiversity in urban green spaces. The ruderal species of Beijing analyzed here comprise 71 native and 49 non-native species, and 90% are of the Cosmopolitan type. Native species accounted for the majority of ruderals across all eight LUTs. Institutional, residential, and woodlot areas with coarser management had higher ruderal species richness than commercial and roadside lawns. Allergenic species showed the highest proportions in municipal parks, and invasive species accounted for 20% of all ruderal species. Urban lawns could support a great number of species without maintenance strategies. The spatial variation in ruderal diversity across the built-up areas was explained best by a combination of land use, soil properties, and social-economic factors. This study urges ecologists, landscape architects, urban managers, and urban dwellers to take into account, without prejudice, common native biological resources and to recognize that urban ruderals have the potential to make significant contributions to building a healthy and sustainable urban green space ecosystem.

## Figures and Tables

**Table 1:** The frequency of ruderal species in different LUTs.

Family	Species	Institutional (%)	Residential (%)	Commercial (%)	Community Park (%)	Municipal Park (%)	Woodlot (%)	Roadside (%)	Riverside (%)
Oxalidaceae	*Oxalis corniculata*	36.3	28.3	24.3	28.6	32.5	6.5	20.3	13.4
Gramineae	*Setaria viridis*	17.6	43.8	27.0	33.3	37.3	64.5	25.0	53.6
Violaceae	*Viola philippica*	14.7	17.2	—	14.3	16.6	—	6.3	14.4
Violaceae	*Viola pekinensis*	13.7	19.3	10.8	—	20.5	25.8	11.5	8.2
Chenopodiaceae	*Chenopodium glaucum*	8.8	27.5	18.9	21.4	25.3	67.7	13.0	26.8
Asteraceae	*Taraxacum mongolicum*	7.8	15.0	13.5	40.5	14.0	12.9	4.7	4.1
Asteraceae	*Inula japonica*	7.8	11.6	2.7	4.8	11.7	16.1	5.2	—
Gramineae	*Poa pratensis*	9.8	11.2	18.9	14.3	10.1	12.9	7.3	2.1
Gramineae	*Digitaria sanguinalis*	5.9	13.7	8.1	28.6	13.6	9.7	23.4	26.8
Asteraceae	*Conyza canadensis*	4.9	6.4	10.8	9.5	2.9	12.9	2.1	2.1
Euphorbiaceae	*Acalypha australis*	4.9	5.2	5.4	9.5	8.1	3.2	6.8	5.2
Amaranthaceae	*Amaranthus retroflexus*	—	10.3	2.7	7.1	3.6	9.7	6.8	9.3
Asteraceae	*Ixeridium sonchifolium*	2.9	11.2	13.5	4.8	7.1	16.1	3.1	1.0
Plantaginaceae	*Plantago depressa*	3.9	7.3	5.4	11.9	14.6	16.1	3.1	4.1
Portulacaceae	*Portulaca oleracea*	—	10.3	10.8	9.5	14.9	—	13.5	7.2
Solanaceae	*Solanum nigrum*	2.9	10.7	8.1	16.7	7.1	19.4	3.6	7.2
Euphorbiaceae	*Euphorbia humifusa*	2.9	4.3	2.7	4.8	5.2	3.2	8.9	9.3
Gramineae	*Eleusine indica*	1.0	15.5	8.1	14.3	13.0	12.9	13.0	—
Rubiaceae	*Rubia cordifolia*	2.9	6.0	—	8.1	8.1	35.5	3.1	14.4
Labiatae	*Leonurus artemisia*	1.0	6.0	—	14.3	5.5	—	4.7	9.3
Asclepiadaceae	*Cynanchum chinense*	1.0	6.9	2.7	—	9.0	16.1	2.6	9.3
Gramineae	*Buchloe dactyloides*	—	4.7	2.7	—	3.6	—	9.9	6.2
Convolvulaceae	*Pharbitis nil*	—	2.6	—	2.4	1.3	—	2.6	11.3
Convolvulaceae	*Calystegia hederacea*	2.0	1.7	5.4	—	4.9	6.5	3.1	10.3

## References

[1] Ohsawa M., Da L.J., Otuka T., Obara H. (1988). Urban vegetation-its structure and dynamics. Integrated Studies in Urban Ecosystems as the Basis of Urban Planning.

[2] Iuliana P.P.U.A., Adelina D., Valentin S., Doina C. (2011). Ecological and aesthetic role of spontaneous flora in urban sustainable landscapes development. J. Plant Dev..

[3] Robinson S.L., Lundholm J.T. (2012). Ecosystem services provided by urban spontaneous vegetation. Urban Ecosyst..

[4] Tredici P.D. (2010). Spontaneous Urban Vegetation: Reflections of Change in a Globalized World. Nat. Cult..

[5] Fischer L.K., Lippe M.V.D., Rillig M.C., Kowarik I. (2013). Creating novel urban grasslands by reintroducing native species in wasteland vegetation. Biol. Conserv..

[6] Mckinney M.L. (2002). Urbanization, biodiversity, and conservation. Bioscience.

[7] Cervelli E.W., Lundholm J.T., Du X. (2013). Spontaneous urban vegetation and habitat heterogeneity in Xi’an, China. Landsc. Urban Plan..

[8] Leung G.P.C., Hau B.C.H., Corlett R.T. (2008). Exotic plant invasion in the highly degraded upland landscape of Hong Kong, China. Biodivers. Conserv..

[9] Wesche K., Krause B., Culmsee H., Leuschner C. (2012). Fifty years of change in Central European grassland vegetation: Large losses in species richness and animal-pollinated plants. Biol. Conserv..

[10] Kowarik I., Marzluff J.M., Shulenberger E., Endlicher W., Alberti M., Bradley G., Ryan C., Simon U., ZumBrunnen C. (2008). On the Role of Alien Species in Urban Flora and Vegetation. Urban Ecology: An International Perspective on the Interaction Between Humans and Nature.

[11] Wania A., Kühn I., Klotz S. (2006). Plant richness patterns in agricultural and urban landscapes in Central Germany—Spatial gradients of species richness. Landsc. Urban Plan..

[12] Muratet A., Porcher E., Devictor V., Arnal G., Moret J., Wright S., Machon N. (2008). Evaluation of floristic diversity in urban areas as a basis for habitat management. Appl. Veg. Sci..

[13] Audrey M., Nathalie M., Frédéric J., Jacques M., Emmanuelle P. (2007). The Role of Urban Structures in the Distribution of Wasteland Flora in the Greater Paris Area, France. Ecosystems.

[14] Rink D. (2009). Wilderness: The Nature of Urban Shrinkage? The Debate on Urban Restructuring and Restoration in Eastern Germany. Nat. Cult..

[15] Prentis E., Norton G.A. (1992). “MEADOWS”—An expert system for the establishment of diverse wildflower grasslands on derelict land in urban areas in the UK. Ecol. Eng..

[16] Kühn N. (2006). Intentions for the Unintentional. J. Landsc. Archit..

[17] Muratet A., Pellegrini P., Dufour A.B., Arrif T., Chiron F. (2015). Perception and knowledge of plant diversity among urban park users. Landsc. Urban Plan..

[18] Hope D., Gries C., Zhu W., Fagan W.F., Redman C.L., Grimm N.B., Nelson A.L., Martin C., Kinzig A. (2003). Socioeconomics drive urban plant diversity. Proc. Natl. Acad. Sci. USA.

[19] Turner K., Lefler L., Freedman B. (2005). Plant communities of selected urbanized areas of Halifax, Nova Scotia, Canada. Landsc. Urban Plan..

[20] Lundholm J.T., Marlin A. (2006). Habitat origins and microhabitat preferences of urban plant species. Urban Ecosyst..

[21] Jim C.Y., Chen W.Y. (2011). Bioreceptivity of buildings for spontaneous arboreal flora in compact city environment. Urban For. Urban Green..

[22] Chen X., Wang W., Liang H., Liu X., Da L. (2014). Dynamics of ruderal species diversity under the rapid urbanization over the past half century in Harbin, Northeast China. Urban Ecosyst..

[23] Merunková K., Chytrý M. (2012). Environmental control of species richness and composition in upland grasslands of the southern Czech Republic. Plant Ecol..

[24] Krasniqi S., Kopali A., Rota E. (2013). Study of plant diversity in the river upstream of Prizreni Lumbardh. Alban. J. Agric. Sci..

[25] Budeanu O. (2013). Management and assessment of medicinal plants from spontaneous flora in the Republic of Moldova. Bull. Transilv. Univ. Bras..

[26] Miller J.R. (2005). Biodiversity conservation and the extinction of experience. Trends Ecol. Evol..

[27] Guo P., Yuebo S.U., Wan W., Liu W., Zhang H., Sun X., Ouyang Z., Wang X. (2018). Urban Plant Diversity in Relation to Land Use Types in Built-up Areas of Beijing. Chin. Geogr. Sci..

[28] He J.Q. (2011). Exotic Plants in China.

[29] He S.Y., Xing Q.H., Yin Z.T., Jiang X.F. (1984). Flora of Beijing.

[30] Compilation Committee of the Flora of China (2004). Flora of China.

[31] Wu Z.Y. (1991). The areal-types of Chinese genera of seed plants. Acta Bot. Yunnanica.

[32] Wan F.H., Liu Q.R., Xie M. (2012). Blological Invasions: Color Illustrations of Invasive Alien Plants in China.

[33] Liu Q.R., Yu M., Zhou Y.L. (2002). Study of alien plants in Beijing. J. Beijing Norm. Univ. (Nat. Sci.).

[34] Investigational Team on Airborne and Allergenic Pollen Grains in China (1991). An Investigation on Airborne and Allergenic Pollen Grains in China.

[35] Beijing Municipal Bureau of Statistics (2014). Beijing Statistical Yearbook.

[36] Braak C.J.F.T., Smilauer P. (2002). CANOCO Reference Manual and CanoDraw for Windows User’s Guide: Software for Canonical Community Ordination (Version 4.5).

[37] Chen K.X. (2005). Study on Types, Distribution Pattern and Seasonal Change of Weed Communities in Urban Ecosystem of Shanghai.

[38] Li J.H. (2007). The Medieinal Plants of Landscape Greening in Northeast Cities. Ph.D. Thesis.

[39] Stewart G.H., Ignatieva M.E., Meurk C.D., Buckley H., Horne B., Braddick T. (2009). URban Biotopes of Aotearoa New Zealand (URBANZ) (I): Composition and diversity of temperate urban lawns in Christchurch. Urban Ecosyst..

[40] Zhao J., Ouyang Z., Hua Z., Zhou W., Wang X., Xu W., Ni Y. (2010). Erratum to: Plant species composition in green spaces within the built-up areas of Beijing, China. Plant Ecol..

[41] Meurk C.D., Sullivan J., Mcwilliam W. (2016). Vegetation History and Dynamics in New Zealand: Future Scenarios and Improved Trajectories Towards Restoring Natural Patterns. Vegetation Structure and Function at Multiple Spatial, Temporal and Conceptual Scales.

[42] Batten L.A. (1972). Breeding Bird Species Diversity in Relation to Increasing Urbanisation. Bird Study.

[43] Livingston M., Shaw W.W., Harris L.K. (2003). A model for assessing wildlife habitats in urban landscapes of eastern Pima County, Arizona (USA). Landsc. Urban Plan..

[44] Flory S., Clay K. (2010). Non-native grass invasion alters native plant composition in experimental communities. Biol. Invasions.

[45] Davies K.W. (2011). Plant community diversity and native plant abundance decline with increasing abundance of an exotic annual grass. Oecologia.

[46] Keane R.M., Crawley M.J. (2002). Exotic plant invasions and the enemy release hypothesis. Trends Ecol. Evol..

[47] Bormann F.H., Balmori D., Geballe G.T., Vernegaard L. (2001). Redesigning the American Lawn: A Search for Environmental Harmony.

